# Optimization of the adsorption and removal of Sb(iii) by MIL-53(Fe)/GO using response surface methodology†

**DOI:** 10.1039/d1ra08169a

**Published:** 2022-02-02

**Authors:** Xiuzhen Yang, Haolin Zhang, Shuangchan Cheng, Bin Zhou

**Affiliations:** College of Civil Engineering, Hunan University of Science and Technology Xiangtan 411201 China yxzhy1102@126.com +86 18073165540

## Abstract

In this study, a graphene oxide metal–organic framework (MIL-53(Fe)/GO) composite adsorbent was successfully synthesized using a simple method at room temperature. The specific surface area of the synthesized MIL-53(Fe)/GO nanoparticles was 268.43 m^2^ g^−1^, with an average pore size of 2.52 nm. The Box–Behnken response surface method was applied to optimize the adsorption time, dosage, pH, temperature, and initial concentration of Sb(iii) in the MIL-53(Fe)/GO adsorption treatment employed for synthetic wastewater containing Sb(iii). We determined the optimal adsorption conditions and explored the isotherm model, adsorption kinetic model, and adsorption mechanism during the adsorption process. For an optimal adsorption of Sb(iii) by MIL-53(Fe)/GO, the adsorption time, dosage, pH, temperature, and initial Sb(iii) concentration should be set to 4.86 h, 85.79 mg L^−1^, 10.00, 39.29 °C, and 10.09 mg L^−1^, respectively. Under these optimal conditions, the removal rate of Sb(iii) will be as high as 97.97%. The adsorption of Sb(iii) by MIL-53(Fe)/GO conformed to the Freundlich isotherm adsorption model, and its maximum adsorption capacity was 69.014 mg g^−1^. The adsorption kinetics process, which is a nonhomogeneous reaction, could be fitted using a quasi-first-order kinetic model. A Fourier transform infrared spectroscopy analysis showed that MIL-53(Fe)/GO hydroxyl and amine groups play a vital role in the adsorption process. MIL-53(Fe)/GO did not exhibit any changes in its adsorption efficiency in the presence of its anion and showed high specificity to Sb(iii). XPS characterization showed that Sb successfully adsorbed onto the adsorbent and that no oxidation–reduction reaction occurred during the adsorption process. The adsorption efficiency remained high even after four cycles of use. MIL-53(Fe)/GO is highly recyclable with significant application potential for treating wastewater containing Sb(iii).

## Abstract 2

In this study, a graphene oxide metal–organic framework (MIL-53(Fe)/GO) composite adsorbent was successfully synthesized using a simple method at room temperature.

## Graphical abstract

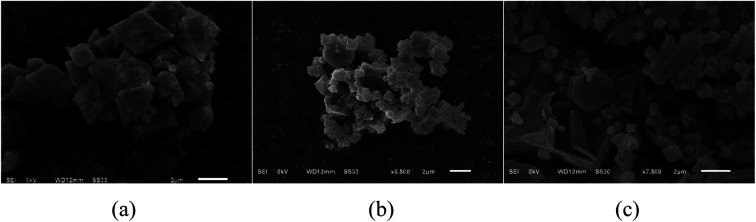

## 1. Introduction

Antimony and its compounds are widely used as flame retardants, polymerization catalysts, and pigments; however, it is highly toxic. The toxicity of Sb(iii) is approximately 10 times that of Sb(v),^1,2^ and long-term exposure to antimony can cause pneumoconiosis, emphysema, and myocardial degeneration.^3^ Severe damage to human DNA^4^ can cause damage to the lungs, heart, and liver, increasing the risk of cancer.^5,6^ Therefore, the European Union, the U.S. Environmental Protection Agency, and other organizations have listed antimony as a pollutant.^7^ China has one of the largest antimony mines in the world. Antimony is being mined in large quantities, and its usage is increasing year by year. From the initial 20 000 tons, China surpassed the United States, Japan, and other countries in 2004, becoming the world's largest consumer of antimony. Unfortunately, antimony is being discharged into the natural environment, causing pollution of the atmosphere, water, plants, and soil.^8^ Therefore, many countries and regions have adopted certain measures to control and limit its content in the natural environment.^7^ For example, the maximum allowable concentration of antimony in drinking water is 2 μg L^−1^ as per Japanese standards, whereas it is 5 μg L^−1^ in Europe.^9^ As per China's surface water quality standards, the antimony concentration in a water source should not exceed 5 μg L^−1^.^10^ Thus, removing Sb(iii) from water bodies is an essential and urgent task.

The adsorption method is a cost-effective water treatment technique, widely used owing to its high adsorption capacity, high efficiency, and adsorbent recyclability. Metal–organic frameworks (MOFs) are a new class of highly porous materials. Owing to their large specific surface area, adjustable pore structure, and many other advantages, they have applications in various fields, including gas storage separation,^11,12^ selective catalysis,^13^ magnetic separation,^14^ chemical sensing,^15^ and drug delivery,^16^ and thus have attracted wide attention.^17^ However, MOFs have certain drawbacks when it comes to their application prospects. To improve their stability and dispersion power, different types of groups have been introduced into porous MOFs. For example, a composite containing MOFs and graphene has been widely researched. MOFs can grow on graphene sheets. Owing to the coordination of the GO oxygen group and the central metal in an MOF, a strong chemical bond and new micropores are formed. Thus, this material exhibits good thermal stability and flexibility^18^ and is often used for photocatalysis^19^ and electrochemistry.^20^ However, there have been no reports on its use for removing heavy metals from water.

## 2. Materials and methods

### 2.1 Preparation of MIL-53(Fe)/GO

First, by applying an ultrasonic treatment for 150 min, a certain quality of GO was uniformly dispersed in 10 mL of DMF at 100 power. We mixed FeCl_3_·6H_2_O, H_2_BDC, and DMF at a molar ratio of 1 : 1 : 280, magnetically stirred for 1 h to make it a clear solution, then added the ultrasonically treated GO/DMF mixture to the above solution, and continued the stirring for 1 h. Subsequently, we transferred it to a 100 mL Teflon liner. Thereafter, the Teflon liner was sealed in a stainless steel autoclave and heated to 150 °C for 20 h. The suspension was obtained by centrifugation and then washed with DMF and C_2_H_5_OH repeatedly by centrifugation. Finally, the solid was dried in vacuum at 80 °C for 10 h to obtain a pale-yellow powder.

### 2.2 Reagent detection and determination

Potassium antimony tartrate was used to prepare a standard stock solution with a Sb(iii) concentration of 1000 mg L^−1^ for subsequent tests. The reagents used in the test were all analytically pure, and the test water was deionized water. A flame atomic absorption spectrophotometer (AA7002A, Beijing Sanxiong Technology Company) was used to determine the concentration of Sb(iii) in the solution. The formula *q* = (*C*_0_ − *C*_e_)/*C*_0_ × 100% was used to calculate the removal rate of Sb, where *q* is the removal rate, %, and *C*_0_ and *C*_e_ are the concentrations of Sb(iii) before and after the adsorption, mg L^−1^, respectively.

### 2.3 Response surface method for optimizing adsorption reaction

The response surface method is an effective method to optimize the process parameters, reduce the number of experiments, and evaluate the level and interaction between the various influencing factors.^21^ Because of the interaction between various factors, conventional single-factor tests do not produce stable and efficient test results.

Box–Behnken design (BBD) is an incomplete three-level factorial design. It is considered an effective technique owing to the number of runs that can be reduced compared with the full-factorial three-level design (FFD). The BBD design does not contain any experimental points at the corner of the cubic surface. This is because when the factors are the same, there is no axial point. On this basis, the response surface method was used to optimize the process of MIL-53(Fe)/GO adsorption of Sb(iii), taking the adsorption time, dosage, pH, temperature, and Sb(iii) initial concentration as the influencing factors. Taking the removal rate of Sb(iii) as the response value, a quadratic polynomial model between the removal rate and various factors was established to obtain the best experimental conditions for the removal of Sb(iii) by adsorption.

The Box–Behnken combination design method in Design-Export software was used to optimize the experiment. We take the optimal value point (0) of each test single factor as the center and the high (+1) and low (−1) levels in the upper and lower regions for the response surface test design: time, dosage, pH, and temperature. The initial concentration of Sb(iii) is the influencing factor, and the adsorption removal rate is the response value. The effects of the first, quadratic (interaction terms), and square terms (surface action) on the adsorption effect were investigated. Forty-six groups of optimization experiments were conducted, six groups of central point experiments were repeated, each group of experiments was repeated thrice, and the average value was taken as the corresponding response value. Table 1 presents the relationship between the three level codes of the five factors and the experimental values. The volume of the solution in the experiment is 50 mL.

**Table 1:** Influencing factors and levels of Box–Behnken design

Factor	Coding	Unit	Level coding value of each factor		
			−1	0	1
Adsorption time	*A*	h	4	5	6
Adsorbent dosage	*B*	mg L^−1^	70	80	90
pH	*C*		8	9	10
Temperature	*D*	°C	25	35	45
Sb(iii) initial concentration	*E*	mg L^−1^	10	20	30

### 2.4 Adsorption isotherm fitting

The formula *Q*_e_ = (*C*_0_ − *C*_e_) × *V*/*m* was used during the test to calculate the adsorption capacity. Here, *C*_0_ and *C*_e_ are the concentrations of Sb(iii) before and after the adsorption, mg L^−1^, respectively; *V* is the volume of the Sb(iii) solution, mL; *m* is the adsorbent dosage, mg.

#### Isothermal adsorption test

We prepared 50 mL of Sb(iii) solution with different concentration gradients (10–180 mg L^−1^), carried out an adsorption test under the best conditions obtained in the response surface optimization experiment, measured the Sb(iii) concentration, and calculated the adsorption capacity *q*_e_. The Langmuir, Freundlich, and Temkin (T–M) isotherm adsorption models were used, as expressed in eqn (1)–(3), respectively, to fit the test results.1
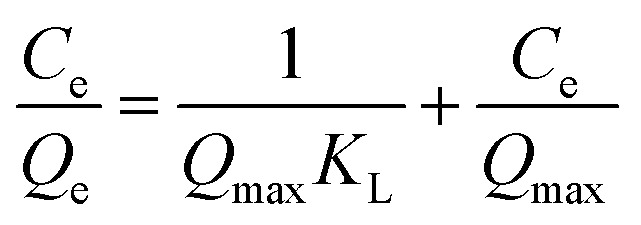
2
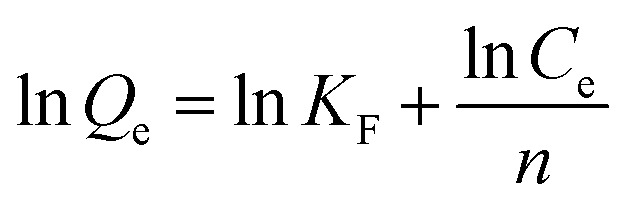

3 *Q*_e_ = *B* ln *A* + *B* ln *C*_e_

where *C*_e_ is the Sb(iii) concentration after adsorption equilibrium, mg L^−1^; *Q*_e_ and *Q*_max_ are the equilibrium adsorption capacity and maximum adsorption capacity, respectively, mg g^−1^; *K*_L_ is the adsorption constant of the Langmuir isotherm adsorption model, L mg^−1^; *K*_F_ is the Freundlich isotherm adsorption model constant, L g^−1^; *n* is the Freundlich isotherm adsorption model constant, dimensionless; *A* and *B* are the Temkin isotherm constants, the values of which can be obtained from the linear relationship in the *Q*_e_–ln *C*_e_ plot.

### 2.5 Adsorption kinetic equation fitting

We prepared 50 mL of Sb(iii) (=10 mg per L of Sb) solution and carried out a kinetic adsorption test under optimal conditions, measured the Sb(iii) concentration in the solution at different times after the reaction starts, and calculated the Sb(iii) adsorption capacity. Three adsorption kinetic models (see eqn (4)–(6)) were used to fit the test results, and the rate control and adsorption mechanism of the adsorption reaction were analyzed.

4 *q*_t_ = *q*_e_(1 − exp(−*k*_1_*t*))

5 *q*_t_ = *q*_e_ − *q*_e_/(*k*_2_*q*_e_*t* + 1)

6
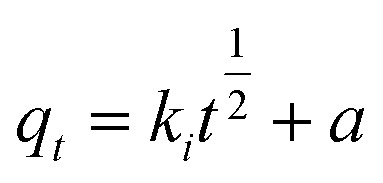
where *q*_*t*_ and *q*_e_ are the adsorption capacity at *t* (min) and adsorption equilibrium, mg g^−1^, respectively; *k*_1_, *k*_2_, and *a* are the adsorption rate constants of the corresponding model, and the units are min^−1^; g (mg min)^−1^ and *k*_*i*_ (g min^0.5^) are the intraparticle diffusion model constant and the adsorption rate constant at a certain stage in the adsorption process.

### 2.6 Characterization method before and after adsorption

An SEM (JSM-6610LV, JEOL, Japan) was used to characterize the morphology of MIL-53(Fe)/GO before and after Sb(iii) adsorption. FT-IR (Nicolet 6700, Thermo Fisher, America) was used to characterize GO/MIL-53 (the functional groups and chemical bond composition before and after Fe adsorption of Sb(iii) were analyzed). XRD (D8-Advance, Bruker, Germany) was used to characterize the MIL-53(Fe)/GO structure. BET (TriStarII 3020, McMuritik, America) was used to measure the specific surface area and pore size of MIL-53(Fe)/GO. XPS (K-Alpha 1063 Thermo Fisher Scientific) was used to analyze the element species in the tested sample, along with the type, content, chemical group, and other information. Finally, the adsorption of Sb(iii) by MIL-53(Fe)/GO was analyzed on the basis of the characterization results.

## 3. Results and discussion

### 3.1 Characterization and adsorption mechanism of MIL-53(Fe)/GO

#### SEM analysis

**Fig. 1:**
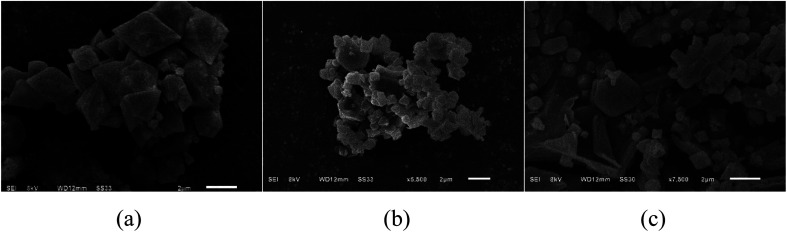
Scanning microscopy images of topography.

Fig. 1 shows the scanning electron micrographs (SEM) of MIL-53 (Fe), MIL-53 (Fe)/GO, and Sb(iii). Fig. 1(a) shows that the size of the MIL-53(Fe) particles is relatively uniform, the surface is smooth and dispersed, and the shape is an octahedral prism structure with sharp ends at the middle.^22^ As shown in Fig. 1(b), MIL-53(Fe)/GO is no longer an angular octahedron, but still maintains the polyhedral structure of MIL-53(Fe), along with crystal size reduction.^23,24^ Although multi-piece bonded, its shape is more disorderly and irregular. GO diffuses densely and tightly on the MIL-53(Fe) surface, which increases the dispersibility of the crystals and increases the specific surface area of the adsorbent. Fig. 1(c) shows that after the adsorption reaction, the material surface is no longer tightly bonded, the skeleton has collapsed, and a large number of amorphous particles are attached onto the material surface, most of which may be adsorbed Sb(iii).

#### XRD characterization

**Fig. 2:**
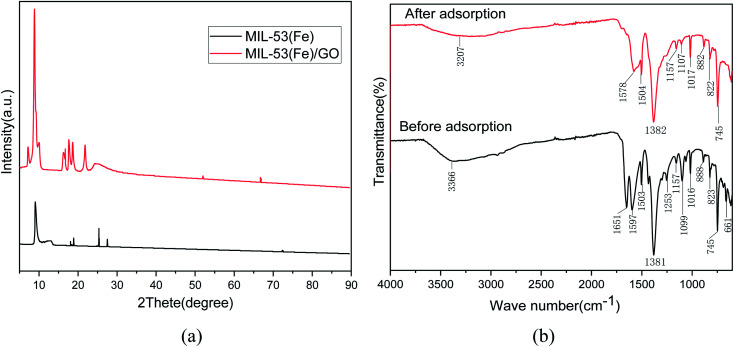
Analysis of XRD and FT-IR spectra before and after Sb(iii) adsorption on MIL-53(Fe)/GO.

The crystal structures of the prepared MIL-53(Fe) and MIL-53(Fe)/GO nanocomposites were characterized by powder X-ray diffraction (Fig. 2(a)). The main diffraction peaks indicate that the high crystallinity of MIL-53(Fe) is consistent with the simulation results.^25,26^ In addition, MIL-53(Fe)/GO and MIL-53(Fe) have similar XRD patterns, indicating that the introduction of GO has no effect on the crystal structure of MIL-53(Fe). However, the intensity of the diffraction peak increases, and a characteristic diffraction pattern of GO can be observed.

#### FT-IR characterization

Fig. 2(b) shows the changes in the Fourier infrared spectrum of MIL-53(Fe)/GO after the adsorption reaction with Sb(iii) in water. In the curve after adsorption, we find that because of the influence of the adsorption reaction, some of the characteristic peaks shift to varying degrees. The characteristic peaks generated by the stretching vibration of hydroxyl (O–H) and (N–H) at 3366 cm^−1^ before adsorption shift to 3207 cm^−1^ after adsorption, *i.e.*, they move toward lower frequencies. This may be due to the replacement of H on the hydroxyl functional group by Sb(iii),^27^ the –C=O group vibration stretching absorption peak may be 1651 cm^−1^, and the surface of the material contains –COOH group^28^ After adsorption, it shifted to 1578 cm^−1^. The characteristic peak produced by the –OH bond at 1099 cm^−1^ shifted to 1107 cm^−1^ after adsorption. This means that the O–H, N–H bond, and hydroxyl (–OH) in the polysaccharide play a major role in the process of Sb(iii) adsorption, and it may be mainly through a complex reaction.^29^ The carboxyl group reacts with Sb(iii).^30^ Therefore, Sb(iii) was successfully adsorbed.

#### Surface area characterization

Typically, in the adsorption reaction process, the larger the specific surface area of a material, the higher its adsorption performance.^31^ Based on the test results of the specific surface area and pore size analysis, the specific surface areas of MIL-53(Fe)/GO and MIL-53(Fe) are 268.43 m^2^ g^−1^ and 194.41 m^2^ g^−1^, respectively. Because graphene oxide is a type of high specific surface area material, after adding graphene oxide, the specific surface area of MIL-53(Fe)/GO is increased. The micropore volumes are 0.12 cm^3^ g^−1^ and 0.05 cm^3^ g^−1^, respectively. Only the micropores in the adsorbent have an adsorption effect. The larger the micropore volume, the better the adsorption effect (Table 2).^32^

**Table 2:** Physical properties of adsorbent

Physical properties	MIL-53(Fe)/GO	MIL-53(Fe)
Specific surface area (m^2^ g^−1^)	268.43	194.41
Average pore size (nm)	2.52	3.74
Micropore volume (cm^3^ g^−1^)	0.12	0.05

**Fig. 3:**
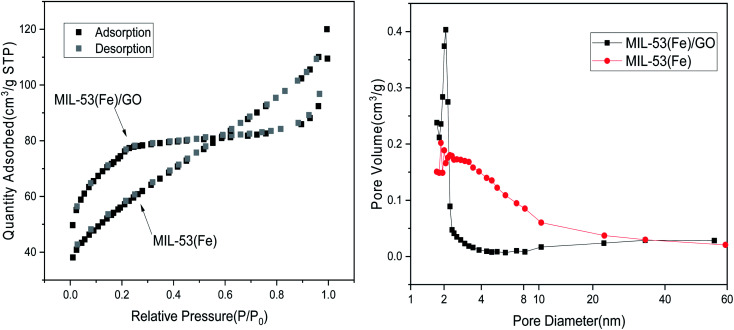
N_2_ adsorption–desorption isotherm.

Fig. 3 shows the N_2_ adsorption/desorption isotherm of MIL-53(Fe)/GO. The sample has H4 curve type IV, which is the main feature of mesoporous materials.^33^ As shown, the pore size is narrowly distributed between 1.72 nm and 2.34 nm, indicating that MIL-53(Fe)/GO has a highly uniform pore structure.^34^

#### XPS characterization

**Fig. 4:**
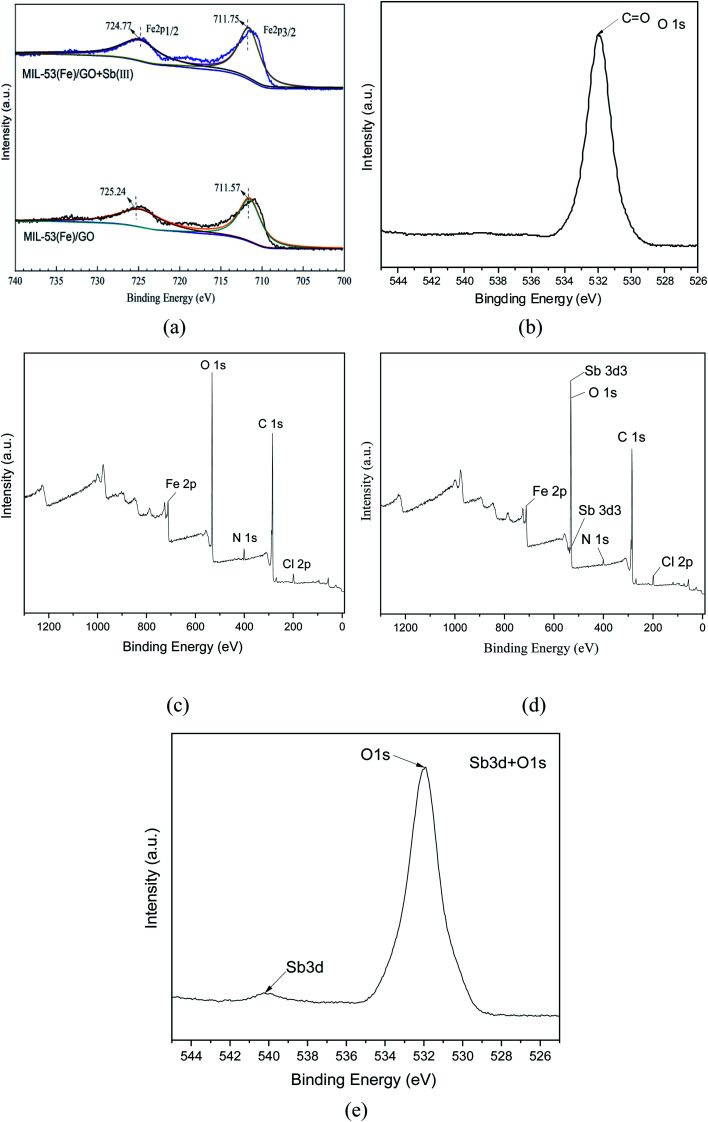
XPS element distributions before and after MIL-53(Fe)/GO adsorption.

X-ray photoelectron spectroscopy (XPS) was used to analyze the elemental composition and electronic structure of the MIL-53(Fe)/GO composite. As shown in Fig. 4(a), there are two peaks in the Fe 2p spectrum. The binding energy peak at 711.57 belongs to Fe 2p_3/2_, and the peak at 725.24 eV belongs to Fe 2p_1/2_. The peak spacing, namely *Δ* = 2p_1/2_ − 2p_3/2_ = 13.67 eV, is consistent with the reported α-Fe_2_O_3_ peak and FeOOH peak.^35,36^ Fe is mainly composed of FeOOH and Fe_2_O_3_ form exists in MIL-53(Fe)/GO. This can be used as a feature of Fe^3+^ in the MIL-53 (Fe) structure.^37^ After MIL-53(Fe)/GO adsorbed Sb(iii), the binding energies corresponding to the Fe diffraction peaks were 711.75 and 724.77 eV, respectively (Fig. 4(a)), indicating that Sb(iii) replaced MIL-53(Fe). The –OH in/GO then forms Fe–O–Sb coordination compound with –O–Fe, so that Sb(iii) is adsorbed.^38^

Fig. 4(b) shows the XPS spectrum of O1s. The spectrum has a peak binding energy of 532.3 eV, which is attributed to the oxygen atom in the carboxylate group of H_2_BDC.^39,40^ These results are similar to the FT-IR spectra, which further confirms the composition of the MIL-53(Fe)/GO composite.

The XPS survey spectrum (Fig. 4(c)) shows the presence of C, N, Cl, O, and Fe elements in MIL-53(Fe)/GO. Fig. 4(d) shows that the Sb peak is added to the spectrum after adsorption and that the position of the Sb peak coincides with the position of O1s. The binding energy of Sb(3d_3_) detected by XPS is 539.9 eV (Fig. 4(e). Based on the chemical state database of XPS, antimony exists in the form of Sb(iii) on the surface of the adsorbent MIL-53(Fe)/GO, and no oxidation–reduction reaction occurs.

### 3.2 Response surface optimization of adsorption conditions

Taking the adsorption time, dosage, pH, temperature, Sb(iii) initial concentration, and other factors as independent variables and the Sb(iii) removal rate as the response value, a response surface quadratic polynomial model is constructed as expressed in eqn (7). The results and analysis of the variance are provided in ESI 1 and 2,† respectively.

7 *Y* = +200.74776 + 10.21229*A* − 0.49821*B* − 32.89125*C* + 1.18421*D* − 2.29854*E* + 0.01950*AB* − 0.47750*AC* − 0.02350*AD* − 0.04250*AE* − 0.08215*BC* − 6.25 × 10^−4^*BD* + 0.0051*BE* − 0.17700*CD* + 0.10100*CE* − 0.01090*DE* − 0.44104*A*^2^ + 0.00861*B*^2^ + 2.72146*C*^2^ + 0.01447*D*^2^ + 0.01229*E*^2^

where *Y* is the response value, %; *A*, *B*, *C*, *D*, and *E* are respectively the adsorption time (h), dosage (mg L^−1^), pH, temperature (°C), initial concentration of Sb(iii) (mg L^−1^), and the actual value corresponding to the independent variable, respectively.

A total of 46 runs were undertaken for optimizing the three individual parameters in the BBD; the experimental conditions based on the factorial design are shown in ESI 1.† The results show that the Sb(iii) removal rate varied in the range of 65.82–97.03%.

From ESI 2,† we find that the model F value is 42.15, *P* < 0.0001, indicating that the nonlinear equation relationship between the respective variables described by the regression equation and the response value is significant; the model determination coefficient *R*^2^ = 0.971, indicating that 2.9% regression equation to explain; *R*_Adj_^2^ − *R*_Pred_^2^ = 0.05 (<0.2), the coefficient of variation CV is 2.06% (<10%), and the signal-to-noise ratio is 25.614 (>4), indicating that the model has high reliability and prediction accuracy and can be used for actual forecast.^41,42^ In addition, from ESI 2,† we find that the adsorption time, dosage pH, temperature, and initial concentration of Sb(iii) significantly affect the removal rate of Sb(iii) (*P* < 0.05). In the interaction term, the interaction between the temperature and pH has a significant effect on the removal rate. The removal rate of Sb(iii) has a significant impact (*P* < 0.05); in the quadratic term, the pH, temperature, and initial concentration have a significant impact on the removal rate of Sb(iii). The *P* value of the other factors is greater than 0.05, which has a significant impact on Sb(iii). The effect of the removal rate is not significant.

**Fig. 5:**
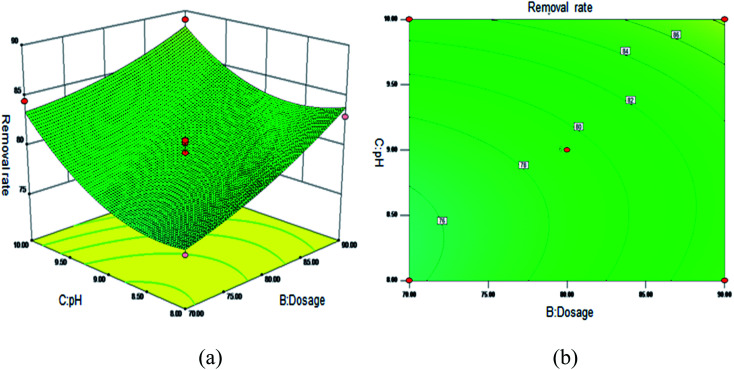
Effect of the interaction between the pH and adsorbent dosage on Sb(iii) removal rate (%). (a) 3D surface plot; (b) contour plot.

**Fig. 6:**
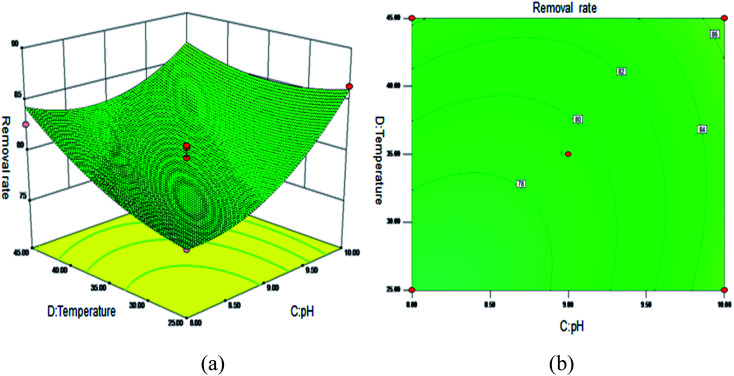
Effect of the interaction between the pH and temperature on Sb(iii) removal rate (%). (a) 3D surface plot; (b) contour plot.

**Fig. 7:**
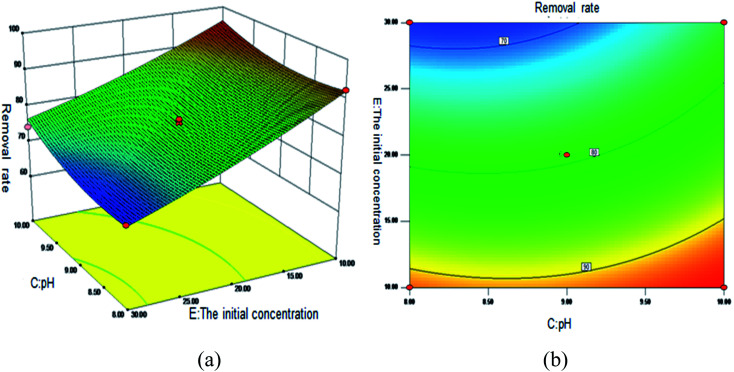
Effect of the interaction between the pH and Sb(iii) initial concentration on Sb removal rate (%). (a) 3D surface map; (b) contour map.

Fig. 5 shows the 3D surface plot and contour plot of the effect of the interaction between the pH value and the amount of adsorbent on the Sb(iii) removal efficiency. Fig. 6 shows the 3D surface plot and contour plot of the effect of the interaction between the pH and temperature on the Sb(iii) removal efficiency. Fig. 7 shows a 3D surface plot and contour plot of the effect of the interaction between the pH and Sb(iii) initial concentration on the Sb(iii) removal efficiency. The results show that when the temperature is in the range of 25–45 °C, the pH value is in the range of 8.0–10, and the dosage is in the range of 70–90 mg, the Sb(iii) removal rate gradually increases. When the initial concentration range of Sb(iii) is 10–30, the Sb(iii) removal rate decreases. This shows that the adsorption efficiency of MIL-53(Fe)/GO on Sb(iii) is proportional to the dosage, temperature and pH value, and inversely proportional to the initial concentration of Sb(iii).

As the amount of adsorbent increases, a large amount of adsorbent can provide more adsorption sites, thereby promoting the adsorption reaction. We speculate the adsorption process to be an endothermic reaction. As the temperature increases, the diffusion coefficient of the adsorbate in the pores can be increased, which is beneficial to the adsorption reaction. As the pH increases, the removal rate increases relatively. When the pH increases to 10, the adsorption rate increases significantly. This is because when pH ≥ 10, antimony exists in the forms of H_2_SbO_3_^−^ and Sb(OH)_4_^−^, and the adsorbent material depletes; protonation makes the negatively charged antimony to easily adsorb under the effect of the electrostatic force. When the initial concentration of the Sb(iii) solution increases from 10 mg L^−1^ to 30 mg L^−1^, the adsorption capacity of MIL-53(Fe)/GO on Sb(iii) increases, but the removal rate decreases. This is because when the quality of the adsorbent MIL-53(Fe)/GO is constant, as the Sb(iii) concentration (III) in the solution increases, the Sb(iii) concentration difference between the solution and the adsorbent increases. This increases the mass transfer driving force between MIL-53(Fe)/GO and Sb(iii)), the adsorbed Sb(iii) enters the active sites on the surface of the adsorbent MIL-53(Fe)/GO more easily, and the adsorption capacity will be greater. However, the active sites on the surface of MIL-53(Fe)/GO are limited; therefore, with the increase in the Sb(iii) concentration (III) in the solution, the removal rate decreases instead.

The first derivative of eqn (7) is calculated to determine the adsorption of Sb(iii) by MIL-53(Fe)/GO. The optimum conditions are as follows: adsorption time 4.86 h, dosage 85.79 mg, pH = 10.00, temperature 39.29 °C, and Sb(iii) initial concentration 10.09 mg L^−1^. Under the best conditions, the removal rate of Sb(iii) by MIL-53(Fe)/GO is 97.97%. To verify the prediction results, the experiment was carried out under optimal conditions. The experiment was repeated thrice, and the average value was taken. The average removal rate of Sb(iii) was 97.60%, which was close to the predicted value of the model (97.97%), and the prediction accuracy reached 99.62%. The predicted value has a high degree of fit with the experimental value, which has a certain guiding significance.

### 3.3 Adsorption isotherm model

**Fig. 8:**
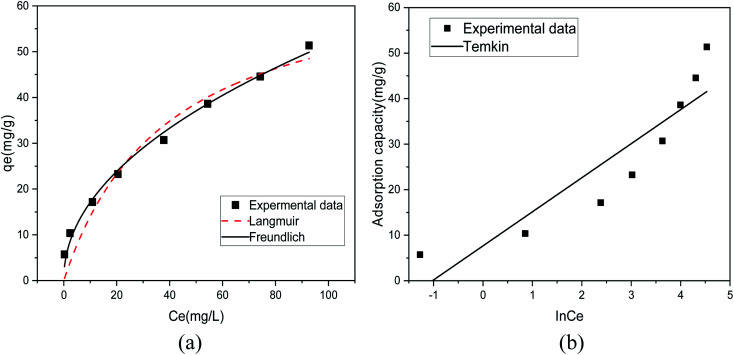
Isothermal model of Sb(iii) adsorption by MIL-53(Fe)/GO.

Fig. 8(a) shows the fitting results of the Langmuir and Freundlich isotherm adsorption models for the adsorption of Sb(iii) by MIL-53(Fe)/GO. Fig. 8(b) shows the fitting results of the T–K isotherm adsorption models. The regression coefficients *R*^2^ of the three isothermal adsorption models are 0.949, 0.991, and 0.827, respectively, indicating that the Freundlich model outperforms the Langmuir model in fitting the adsorption and removal processes of Sb(iii) by MIL-53(Fe)/GO. The Freundlich isotherm model assumes that the adsorption sites of the adsorbent are evenly distributed on the adsorbent surface, which is a multiphase adsorption surface, or active sites are supported on the surface, suitable for single-layer (chemical) or multilayer (physical) adsorption.^42^ Therefore, it is speculated that the adsorption behavior is a heterogeneous reaction.^43,44^ The calculated maximum adsorption capacity *q*_max_ is 69.014 mg g^−1^ (Table 3).

**Table 3:** Adsorption isotherm model parameters for the adsorption of Sb(iii) by MIL-53(Fe)/GO

Model	Langmuir model	Freundlich model	Temkin model
Parameters	*q*_max_ = 69.014	*K*_F_ = 5.642	*A* = 2.773
	*K*_L_ = 0.025	*n* = 2.078	*B* = 7.483
	*R* ^2^ = 0.949	*R* ^2^ = 0.991	*R* ^2^ = 0.827

### 3.4 Adsorption kinetic model

**Fig. 9:**
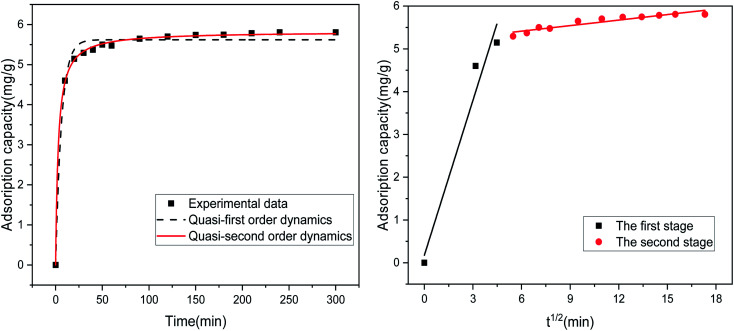
Kinetic model of the adsorption of Sb(iii) by MIL-53(Fe)/GO.

Table 4 lists the results of the fitting-related parameters of the kinetic model for the adsorption of Sb(iii) by MIL-53(Fe)/GO. From Table 4, we find that both the quasi-first-order and quasi-second-order kinetic models can fit the experimental data well. The quasi-first-order kinetic model has a high fitting accuracy (*R*^2^ = 0.987), whereas the theoretical value (*q*_e_ = 5.824 mg g^−1^) fitted by the quasi-second-order kinetic model is close to the experimental value (*q*_e_ = 5.806 mg g^−1^), and the fitting accuracy (*R*^2^ = 0.999) is greater. Therefore, it is inferred that MIL-53(Fe)/GO is relative to Sb(iii) and that the adsorption process follows a quasi-two-stage kinetic model, indicating that the adsorption reaction is mainly chemical adsorption.^43^ To identify the antimony migration process on MIL-53(Fe)/GO crystals, based on kinetic data, the rate control step was analyzed using the intra-particle diffusion model, which characterizes two or more steps involved in the adsorption process.^45–47^Fig. 9 shows that the adsorption process tends to two stages. The adsorption process is carried out by surface adsorption and intra-particle diffusion (antimony is transferred from the boundary membrane to the adsorbent surface and then from the adsorbent surface to the active site or binding site in the particle).^48^ Based on the particle diffusion model, the slope of the linear graph is defined as the diffusion rate constant. The slopes of the fitting lines in the two stages are different, indicating a gradual stage in the adsorption process. The *k*_1_ > *k*_2_ of the two steps means that the adsorption process starts from the beginning. The adsorption rate of external diffusion is the highest, and the current adsorption rate is mainly controlled by pore (in-particle) diffusion rather than boundary layer (external) diffusion.^49,50^

**Table 4:** Adsorption kinetic model parameters related to the adsorption of Sb(iii) by MIL-53(Fe)/GO

Model	Quasi-first-order dynamics model	Quasi-two-stage dynamic model	Intraparticle diffusion model
Parameter	*q*_e_ = 5.620	*q*_e_ = 5.824	*α*_1_ = 0.177
			*k*_1_ = 1.207
	*K*_1_ = 0.157	*K*_1_ = 0.061	*R* ^2^ = 0.963
			*α*_1_ = 5.154
	*R* ^2^ = 0.987	*R* ^2^ = 0.999	*K*_2_ = 0.043
			*R* ^2^ = 0.882

### 3.5 Influence of coexistent ions

**Fig. 10:**
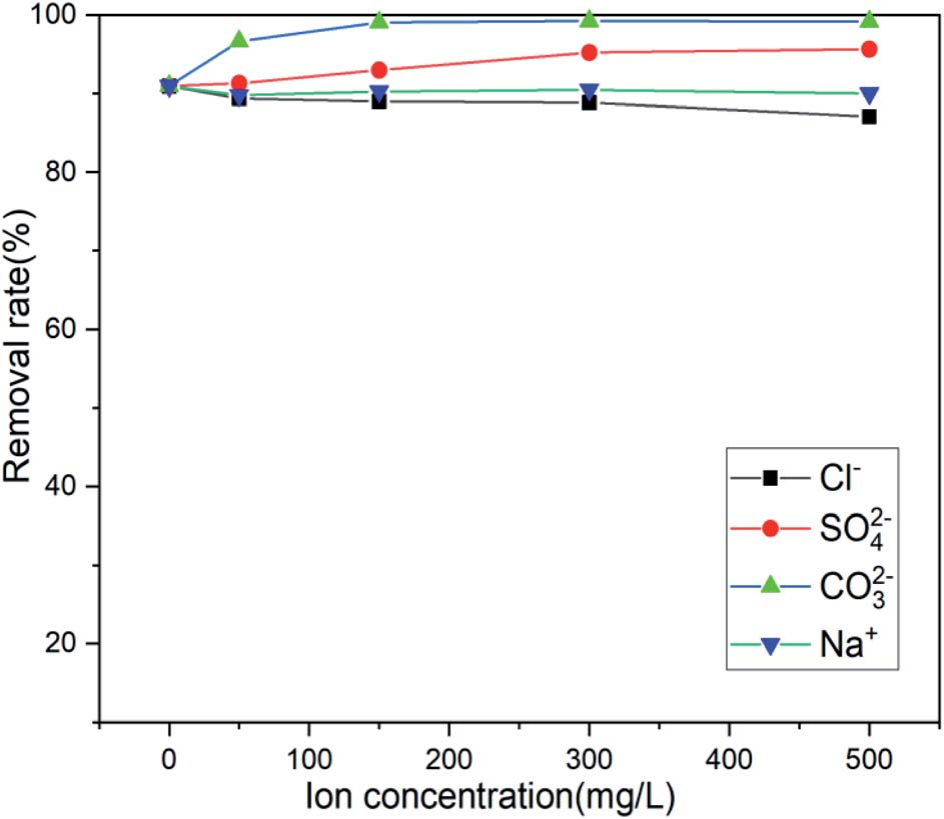
Effect of coexisting ions on adsorption.

Actual wastewater is often complex in terms of its composition. There are multiple anions and different heavy metal cations. Because the adsorption process will compete with the target pollutants to be removed, the existence of coexisting ions is another important factor affecting the actual removal capacity in practice.^51,52^ Several anions coexisting near the mining area were selected (Cl^−^, SO_4_^2−^, and CO_3_^2−^), and the influence of anion concentration on the adsorption efficiency of the adsorbent was investigated. Fig. 10 shows that only Cl^−^ has a slight effect on the adsorption and that SO_4_^2−^ and CO_3_^2−^ promote adsorption, Na^+^ has no effect on its adsorption, which is beneficial to the adsorption of Sb(iii) by the adsorbent in actual wastewater. When Mn^2+^ and Pb^2+^ coexist, the adsorbent will only adsorb Sb(iii), showing a high degree of specificity for Sb(iii).

### 3.6 Recycling of MIL-53(Fe)/GO

**Fig. 11:**
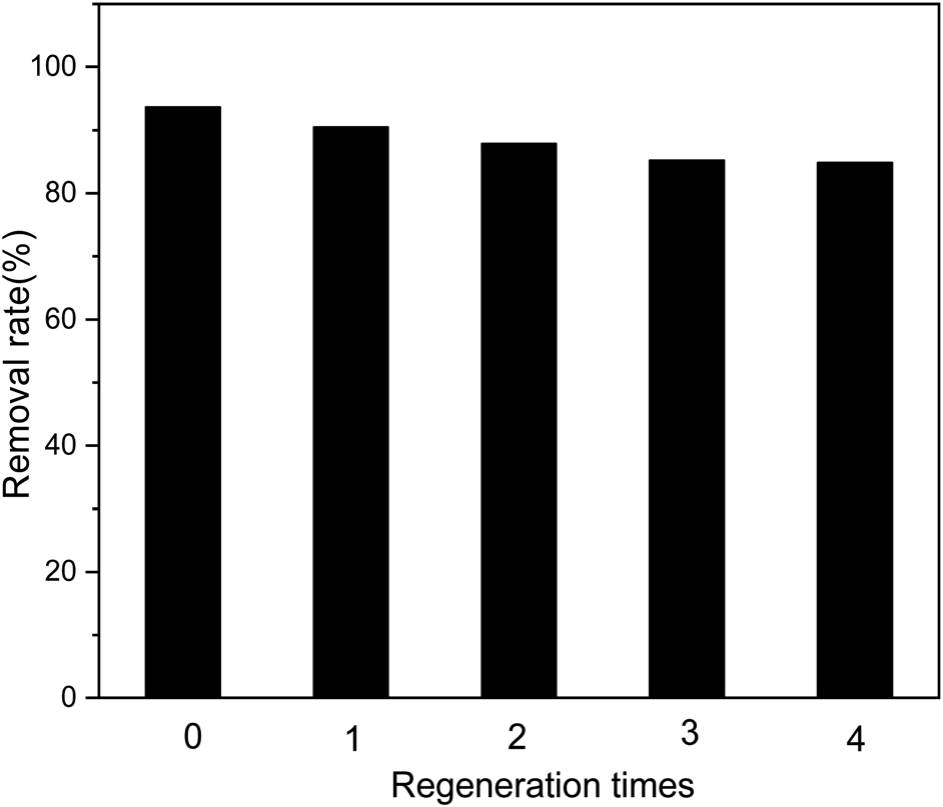
Effect of regeneration time on adsorption efficiency.

The recyclable performance of adsorbents is an important indicator that can help evaluate their economical aspects in practical applications; adsorption materials that can be recycled multiple times are economical. As shown in Fig. 11, after four cycles of use of the adsorbent, the removal efficiency for Sb(iii) in the solution does not decrease significantly.

## 4. Conclusions

The response surface optimization experiments showed that the dosage, pH, temperature, and initial concentration of Sb(iii) significantly affect the adsorption of Sb(iii) by MIL-53(Fe)/GO. The adsorption time had no significant effect on it. For optimal adsorption, the adsorption time, dosage, pH, temperature, and initial concentration of Sb(iii) should be set to 4.86 h, 85.79 mg, 10.00, 39.29 °C, and 10.09 mg L^−1^, respectively. The average removal rate was as high as 97.60%. The Freundlich isotherm model could effectively fit the adsorption process of Sb(iii) by MIL-53(Fe)/GO (*R*^2^ = 0.991). The maximum adsorption capacity was 69.014 mg g^−1^, and the adsorption was a heterogeneous reaction, exhibiting quasi-secondary kinetics. The scientific model could better fit the adsorption kinetic process (*R*^2^ = 0.999). The adsorption process was mainly chemical adsorption, and the adsorption process was carried out through surface adsorption and intraparticle diffusion. The FT-IR analysis showed that the O–H, N–H bond, and hydroxyl (–OH) in the polysaccharide play a major role in the adsorption of Sb(iii) and that the carboxyl group reacts with Sb(iii) chemically. The abundant hydroxyl on the adsorbent surface was the main reason for the excellent adsorption and removal performance of Sb(iii). The hydroxyl group in Fe–O–OH was replaced by Sb(iii) to form a new complex Fe–O–Sb. In the presence of Cl–, SO_4_^2−^, CO_3_^2−^, and Na^+^, the adsorbent still showed superiority in the adsorption of antimony. In the presence of both Mn^2+^ and Pb^2+^, the adsorbent showed specificity to antimony. Even after the fourth cycle of use, it still exhibited a high removal rate, making it a potential adsorbent for removing antimony from water.

## Conflicts of interest

The authors declare no conflict of interest regarding the publication of this paper. We do not have any commercial or associative interest that would have a potential conflict of interest in connection with the work submitted.

## Supplementary Material

RA-012-D1RA08169A-s001
