# Supplementary material for: Optimization of the adsorption and removal of Sb(iii) by MIL-53(Fe)/GO using response surface methodology
Source: RSC Adv. 2022 Feb 2;12(7):4101–12. doi: 10.1039/d1ra08169a (PMC8980999; doi:10.1039/d1ra08169a)
Supplement: RA-012-D1RA08169A-s001 [file RA-012-D1RA08169A-s001.pdf]

Electronic Supplementary Material (ESI) for RSC Advances.
This journal is © The Royal Society of Chemistry 2022

Supplementary Information (SI):

SI 1 Experimental Box–Behnken design and removal rate of Sb(III)

Standard order	Experiment order	Influencing factor coding level					Sb(III)adsorption rate (%)	
		A	B	C	D	E	Actual value	Predictive value
1	41	-1	-1	0	0	0	73.73	75.22
2	30	1	-1	0	0	0	74.57	77.61
3	21	-1	1	0	0	0	82.36	81.36
4	43	1	1	0	0	0	83.98	84.53
5	4	0	0	-1	-1	0	76.39	76.43
6	2	0	0	1	-1	0	86.34	86.04
7	16	0	0	-1	1	0	82.64	84.35
8	14	0	0	1	1	0	85.51	86.88
9	26	0	-1	0	0	-1	91.31	89.43
10	36	0	1	0	0	-1	94.71	94.94
11	37	0	-1	0	0	1	69.39	66.74
12	22	0	1	0	0	1	74.83	74.29
13	28	-1	0	-1	0	0	77.94	76.64
14	32	1	0	-1	0	0	81.80	80.37
15	17	-1	0	1	0	0	85.54	83.66
16	44	1	0	1	0	0	87.49	85.48
17	3	0	0	0	-1	-1	89.68	89.49
18	10	0	0	0	1	-1	97.03	96.05
19	1	0	0	0	-1	1	70.44	70.00
20	9	0	0	0	1	1	73.43	72.20
21	23	0	-1	-1	0	0	75.17	75.73
22	45	0	1	-1	0	0	82.95	83.88
23	18	0	-1	1	0	0	84.50	83.42
24	25	0	1	1	0	0	89.03	88.32
25	6	-1	0	0	-1	0	76.47	76.45
26	7	1	0	0	-1	0	79.28	79.70
27	15	-1	0	0	1	0	82.24	81.29
28	12	1	0	0	1	0	84.11	83.61
29	34	0	0	-1	0	-1	92.21	92.02
30	33	0	0	1	0	-1	93.69	96.07
31	40	0	0	-1	0	1	68.66	68.33
32	35	0	0	1	0	1	74.18	76.41
33	31	-1	0	0	0	-1	87.81	89.06
34	39	1	0	0	0	-1	93.32	92.70
35	42	-1	0	0	0	1	65.82	68.24
36	46	1	0	0	0	1	69.63	70.18
37	8	0	-1	0	-1	0	75.81	76.05
38	5	0	1	0	-1	0	82.46	82.70
39	13	0	-1	0	1	0	80.26	80.55
40	11	0	1	0	1	0	86.66	86.96
41	19	0	0	0	0	0	77.60	79.26
42	20	0	0	0	0	0	78.96	79.26
43	24	0	0	0	0	0	78.84	79.26

44	27	0	0	0	0	0	80.32	79.26
45	29	0	0	0	0	0	79.26	79.26
46	38	0	0	0	0	0	80.56	79.26

SI 2 ANOVA analysis for variance of the model

Parameter	Sum of squares	Degree of freedom	Mean square	F value	P value	
model	2421.56	20	121.08	42.15	< 0.0001	significant
A	31.00	1	31.00	10.79	0.0030	
B	170.56	1	170.56	59.38	< 0.0001	
C	147.14	1	147.14	52.13	< 0.0001	
D	76.61	1	76.61	26.67	< 0.0001	
E	1878.79	1	1878.79	654.12	< 0.0001	
AB	0.15	1	0.15	0.053	0.8199	
AC	0.91	1	0.91	0.32	0.5781	
AD	0.22	1	0.22	0.077	0.7838	
AE	0.72	1	0.72	0.25	0.6204	
BC	2.64	1	2.64	0.92	0.3468	
BD	0.016	1	0.016	0.005	0.9418	
BE	1.04	1	1.04	0.36	0.5527	
CD	12.53	1	12.53	4.36	0.0471	
CE	4.08	1	4.08	1.42	0.2445	
DE	4.75	1	4.75	1.65	0.2101	
A^2	1.70	1	1.70	0.59	0.4492	
B^2	6.48	1	6.48	2.25	0.1457	
C^2	64.64	1	64.64	22.50	< 0.0001	
D^2	18.28	1	18.28	6.36	0.0184	
E^2	13.20	1	13.20	4.60	0.0420	
Residual	71.81	25	2.87			
Lack of Fit	65.97	20	3.30	2.83	0.1263	not significant
Pure Error	5.84	5	1.17			
Cor Total	2493.37					
